# The future of diagnosis in clinical neurosciences: Comparing multiple sclerosis and schizophrenia

**DOI:** 10.1192/j.eurpsy.2023.2432

**Published:** 2023-07-21

**Authors:** Błażej Misiak, Jerzy Samochowiec, Krzysztof Kowalski, Wolfgang Gaebel, Claudio L. A. Bassetti, Andrew Chan, Philip Gorwood, Sergi Papiol, Geert Dom, Umberto Volpe, Agata Szulc, Tamas Kurimay, Hilkka Kärkkäinen, Andre Decraene, Jan Wisse, Andrea Fiorillo, Peter Falkai

**Affiliations:** 1 Department of Psychiatry, Wroclaw Medical University, Wroclaw, Poland; 2 Department of Psychiatry, Pomeranian Medical University, Szczecin, Poland; 3 Department of Psychiatry and Psychotherapy, LVR-Klinikum Düsseldorf, Medical Faculty, Heinrich-Heine-University, Düsseldorf, Germany; 4 WHO Collaborating Centre on Quality Assurance and Empowerment in Mental Health, DEU-131, Düsseldorf, Germany; 5 Department of Neurology, Inselspital, Bern University Hospital, University Bern, Switzerland; 6 Interdisciplinary Sleep-Wake-Epilepsy-Center, Inselspital, Bern University Hospital, University Bern, Bern, Switzerland; 7 Université Paris Cité, INSERM, U1266 (Institute of Psychiatry and Neuroscience of Paris), Paris, France; 8 CMME, GHU Paris Psychiatrie et Neurosciences, Hôpital Sainte-Anne, Paris, France; 9 Centro de Investigación Biomédica en Red de Salud Mental (CIBERSAM), Madrid, Spain; 10 Department of Psychiatry, Institute of Psychiatric Phenomics and Genomics, University Hospital, Ludwig Maximilian University, Munich, Germany; 11 Collaborative Antwerp Psychiatric Research Institute, University of Antwerp, B-2610 Antwerp, Belgium; 12 Multiversum Psychiatric Hospital, B-2530 Boechout, Belgium; 13 Unit of Clinical Psychiatry, Department of Clinical Neurosciences/DIMSC, Polytechnic University of Marche, 60126 Ancona, Italy; 14 Department of Psychiatry, Medical University of Warsaw, Warsaw, Poland; 15 Department of Psychiatry, St. Janos Hospital, Budapest, Hungary; 16 President of GAMIAN-Europe, Ixelles, Belgium; 17 European Federation of Associations of Families of People with Mental Illness (EUFAMI), Leuven, Belgium; 18 Century House, Wargrave Road, Henley-on-Thames, Oxfordshire RG9 2LT, UK; 19 Department of Psychiatry, University of Campania “Luigi Vanvitelli”, Naples, Italy; 20 Department of Psychiatry and Psychotherapy, University Hospital, LMU Munich, Nussbaumstraße 7, 80336 Munich, Germany

**Keywords:** diagnosis, dimension, multiple sclerosis, neurology, psychiatry, psychopathology, reliability, schizophrenia, validity

## Abstract

The ongoing developments of psychiatric classification systems have largely improved reliability of diagnosis, including that of schizophrenia. However, with an unknown pathophysiology and lacking biomarkers, its validity still remains low, requiring further advancements. Research has helped establish multiple sclerosis (MS) as the central nervous system (CNS) disorder with an established pathophysiology, defined biomarkers and therefore good validity and significantly improved treatment options. Before proposing next steps in research that aim to improve the diagnostic process of schizophrenia, it is imperative to recognize its clinical heterogeneity. Indeed, individuals with schizophrenia show high interindividual variability in terms of symptomatic manifestation, response to treatment, course of illness and functional outcomes. There is also a multiplicity of risk factors that contribute to the development of schizophrenia. Moreover, accumulating evidence indicates that several dimensions of psychopathology and risk factors cross current diagnostic categorizations. Schizophrenia shares a number of similarities with MS, which is a demyelinating disease of the CNS. These similarities appear in the context of age of onset, geographical distribution, involvement of immune-inflammatory processes, neurocognitive impairment and various trajectories of illness course. This article provides a critical appraisal of diagnostic process in schizophrenia, taking into consideration advancements that have been made in the diagnosis and management of MS. Based on the comparison between the two disorders, key directions for studies that aim to improve diagnostic process in schizophrenia are formulated. All of them converge on the necessity to deconstruct the psychosis spectrum and adopt dimensional approaches with deep phenotyping to refine current diagnostic boundaries.

## Introduction

Advances in neuroscience have blurred the boundaries between neurology and psychiatry, which have similar roots but had been separated in the first half of the 20th century [1]. In the last few decades, a reapproach between psychiatry and neurology has been taking place for various reasons, including shared neural substrates, high rates of comorbidity and similar treatment approaches. In some countries (e.g. Switzerland), the reapproach between neurology and psychiatry (as well as other clinical neurodisciplines) has been accompanied by the creation of overarching societies and joint journals [2, 3].

Psychiatry and neurology still show major differences in the diagnosis and management of patients. Although the diagnostic process in both specialties remains to be based on medical history and clinical examination, neurology can rely on more specific (bio)markers. This difference originates from substantial advances in understanding pathophysiological processes underlying the development of neurological disorders. Importantly, similarities and differences between neurology and psychiatry can provide grounds for a better understanding of the underlying pathophysiology and improvement of case management [4].

Schizophrenia, one of the most burdensome and costliest mental disorders [5, 6], has recently been conceptualized as a disorder of brain connectivity, with important diagnostic, therapeutic and prognostic implications [7]. However, its diagnosis is still based on the presence and duration of specific psychopathological symptoms and exclusion of conditions that might better explain observed psychopathology. Importantly, schizophrenia shares some similarities with multiple sclerosis (MS), which is perceived as a progressive demyelinating disease of the brain and the spinal cord. Similarities include age of onset, the presence of sex differences, shared risk factors (seasonality of birth and psychosocial stress), involvement of immune-inflammatory processes, neurocognitive impairment, sleep–wake disturbances (including fatigue, sleepiness and insomnia, narcolepsy-like phenotypes) and trajectories of disease course (Table 1). Epidemiological studies show that a diagnosis of MS might be a risk factor for the subsequent development of schizophrenia [8-12]. Furthermore, psychotic symptoms can be the first manifestation of an underlying demyelinating process in MS. However, causal associations remain unclear, although some hypotheses have already been considered, including shared environmental and genetic risk factors, overlapping neuropathological processes and psychosocial stress related to the onset of MS [13]. For instance, there is evidence that MS shares genetic background with schizophrenia, but not with bipolar disorder [14]. These observations might also have certain clinical implications. Indeed, recent systematic reviews of case reports demonstrated that upon a diagnosis of MS, immunosuppressive therapy might be significantly more effective for psychotic symptoms than antipsychotics in this population [15, 16]. In the majority of reviewed cases (60%), fronto-temporal lesions were identified [15]. On the other side, individuals with schizophrenia often show clinical indices of the central nervous system (CNS) damage, called neurological soft signs. Indeed studies show that even 73% of patients perform outside the range of healthy controls on aggregate measures of neurological soft signs that include subclinical impairments of sensory integration, motor coordination and sequencing of complex movements [17, 18].

**Table 1.** Overview of similarities and differences in schizophrenia and MS

	Schizophrenia	MS
Age at onset	Peaks between 21 and 25 years of age in males; between 25 and 30 years of age and after 45 years of age in females [21]	Usually between 20 and 40 years of age [22]
Sex differences	Females tend to show later age of onset, more mood symptoms, less negative symptoms and less severe course of illness [23]	Higher prevalence in females, greater neurological progression in males [24]
Heritability	Around 50–80% [25–27]^a^	Around 3–25% [28–30]^a^
Environmental risk factors	Winter–spring season of birth in northern hemisphere, obstetric complications, psychosocial stress (especially childhood trauma), heavy cannabis use, ethnic minority status, migration, urbanicity and *Toxoplasma gondii* seropositivity [31–33]	Spring seasonality of birth in northern hemisphere, adolescent obesity, Epstein–Barr virus seropositivity, infectious mononucleosis, cigarette smoking and psychosocial stress [24, 34, 35]
Involvement of immune-inflammatory processes	Yes [36]	Yes [37]
Association with specific HLA loci	Yes [14, 38]	Yes [14, 38]
Diagnosis: clinical and biomarker guided	Based on symptomatic manifestation and duration of symptoms. It is necessary to add course and symptom specifiers [20]. Biomarkers: none	Based on symptomatic manifestation [19]. Biomarkers: neuroimaging findings and assessment of CSF. Provision of disease course and specification of disease activity are needed to guide therapy.
Neuroimaging findings	Non-specific, neurodevelopmental and neuroprogressive [39]	Demyelinating white matter lesions on MRI, grey matter involvement, atrophy, microstructural damage also of the so-called normal-appearing white and grey matter [19]
Clinical manifestation	Positive, negative, disorganization and mood symptoms, neurocognitive impairments as well as sleep–wake disturbances [40]	Neurological symptoms, neurocognitive impairments and sleep–wake disturbances; possible co-occurrence with psychosis, mood and anxiety disorders [19, 41]
Course of illness	Single or multiple episodes with full or partial recovery, chronic course [40]	Initially relapsing, with possible development into (secondary) progressive course, minority (approx. 15% primary progressive from onset) [42]
Pharmacological treatment	Targeting neurotransmission [43]	Targeting aberrant immune-inflammatory responses [44]
Effectivity of treatment	Recovery rate of 24% [45]^b^	Relapse recovery rate of 50–65% [46]

Abbreviations: HLA, human leukocyte antigen.

a Based on concordance rates in monozygotic and dizygotic twins.

b Higher recovery rate (32–38%) in case of individuals with first-episode psychosis [47, 48].

The diagnostic process in MS is largely different compared to that of schizophrenia. Apart from clinical symptoms, current diagnostic criteria emphasize the role of neuroimaging examination, ophthalmologic findings and assessment of visual evoked potential and cerebrospinal fluid (CSF) [19]. However, certain important similarities need to be acknowledged. Although both the International Classification of Diseases (ICD-11) and the *Diagnostic and Statistical Manual of Mental Disorders* (DSM-5) systems have abandoned the necessity to identify the type of schizophrenia, it is still needed to add course and symptom specifiers [20]. Similarly, at the time of MS diagnosis, a provisional disease course (relapsing–remitting, primary progressive or secondary progressive), and more specifically whether the course is active or not, and progressive or not, according to the preceding year’s history should be specified [19].

Taking these considerations into account, it might be concluded that both schizophrenia and MS share some similarities, yet they largely differ in terms of diagnostic process. The major difference is that the diagnostic criteria of MS also include paraclinical markers. This difference is likely attributable to a better understanding and availability of accurate markers in case of MS. Therefore, in the present article, the current state of diagnostic process of schizophrenia was critically reviewed. Advances in the management of MS based on sound pathophysiological insights into neurobiology-based diagnostic process, treatment strategies and monitoring were considered in order to highlight the future prospects for improvement of diagnostic process in schizophrenia.

## Diagnosis in psychiatry and neurology: The case of schizophrenia and MS

### Reliability of diagnosis

Reliability is usually measured as the extent of agreement between assessors examining the same subjects [49]. Results of reliability analysis can be expressed as the kappa statistic referring to the percentage of subjects in which agreement on specific variables is reached [50]. The kappa values can be interpreted as slight (0-0.20), fair (0.21-0.40), moderate (0.41-0.60), substantial (0.61-0.80) and almost perfect (0.81-1.0) [51].

Field trials for the DSM-5 estimated the kappa statistic for schizophrenia diagnosis at 0.46, showing moderate reliability [52]. By contrast, field studies for ICD-11 revealed the kappa statistic of 0.87 for a diagnosis of schizophrenia that can be interpreted as “almost perfect” [53]. These differences are likely attributable to methodological differences across field studies of the ICD-11 and DSM-5 criteria. Indeed, field studies for the DSM-5 criteria did not use structured interviews and were based on a sequential test–retest design (two raters interviewing the participant at two different time points). However, a recent systematic review and meta-analysis comparing kappa statistics for schizophrenia, schizoaffective disorders, unipolar depression and bipolar disorder reported that the type of diagnostic interview and diagnostic manual as well as the methods used to calculate the kappa statistics did not account for heterogeneity of results [54]. Interestingly, the authors found that the kappa statistic was substantially lower for schizoaffective disorder (0.57) compared to that for schizophrenia (0.80), bipolar disorder (0.82) and unipolar depression (0.75).

The diagnosis of MS has been much improved in the last decades by the systematic use of neuroimaging and CSF biomarkers and the definition of clear diagnostic guidelines [19, 55]. Today an early and accurate diagnosis has become possible and essential for the appropriate use of an extensive therapeutic armamentarium, which has significantly improved the prognosis of MS, the prevention of disabling forms of the condition and eventually patients’ health-related quality of life [56]. Further diagnostic progresses are expected in near future by the use of refined neuroimaging approaches (e.g. imaging of the central veins within MS lesions, meningeal B cell aggregates, quantitation of grey matter and spinal cord pathology) and serum biomarkers (e.g. neurofilament measurement as surrogate markers of neuronal loss) [56].

### Validity of diagnosis

There is no single and uniform definition of validity, and some authors even refer to philosophical concepts of “realism” in order to better explain the core aspects underlying validity of diagnosis. According to the most recent and simplistic definitions, validity of diagnosis is “the proportion of the total variance of the diagnosis due to disorder” [57]. In other words, validity of diagnosis reflects the extent by which the diagnosis explains the presence or absence of the disorder in a specific population, i.e., “the nature of reality” [58].

However, one of the most important limitations of the validity construct in psychiatry is the impossibility to claim about the presence or absence of the disorder without reference to diagnosis. In this regard, validity of psychiatric diagnoses must be addressed from various perspectives. Therefore, Robins and Guze [59] were the first to propose the ways validity of psychiatric diagnosis can be tested. The authors proposed five aspects that need to be investigated to inform about the validity of psychiatric diagnoses, including clinical description, laboratory studies, exclusion criteria, follow-up studies and family studies. Subsequently, Kendler [60] proposed to investigate three subgroups of variables: (1) antecedent validators (family history of disease, premorbid personality and precipitating factors), (2) concurrent validators (psychological tests and assessment), (3) predictive validators (diagnostic stability over time, risk of relapse, recovery rates and response to treatment). With ongoing progress of neurosciences, the list of potential aspects of validity might include molecular genetics, molecular biology, neurochemistry, neuroanatomy, neurophysiology and cognition [61].

Among available indicators, diagnostic stability is the most direct measure of validity [62]. It is expressed as the percentage of patients consistently diagnosed as meeting the criteria of a specific disorder over time. A recent systematic review demonstrated mean diagnostic stability for schizophrenia of 84.3% and 67.1% for prospective and retrospective studies, respectively [63]. The following factors were associated with greater diagnostic stability: male sex, older age at study inception, older age at onset, late stages of illness, family history of mental illness, poorer functioning and longer length of hospital stay.

It should be noted that current approaches to validity neglect the possibility that psychiatric disorders, including schizophrenia, are not categorical constructs, and thus clear diagnostic boundaries cannot be established [64, 65]. Indeed, there is mounting evidence that schizophrenia is characterized by high inter-individual variability in terms of several characteristics, including premorbid functioning, psychopathological manifestation, extent of cognitive impairment, symptomatic response to treatment and functional outcomes. Apart from clinical heterogeneity, low validity of schizophrenia diagnosis can also be reflected by high comorbidity rates with other psychiatric diagnoses, e.g., schizotypal personality disorder or substance use disorders [66, 67]. Also, antipsychotics that are the mainstay treatment of schizophrenia appear to be effective in case of other mental disorders, including mood disorders [67]. Moreover, the pathophysiology of schizophrenia remains largely unknown. This complexity is also reflected by studies showing that schizophrenia shares genetic background with other mental disorders [68–70]. Therefore, it is now intensively investigated as to whether a dimensional approach might better explain variations in psychopathology. Interesting findings from the Bipolar-Schizophrenia Network for Intermediate Phenotypes (B-SNIP) study support this approach. The authors analysed the extent of mood and psychotic symptomatology in 762 patients with psychotic disorders and found that 45% of cases fall in the continuum between categorical diagnostic constructs of schizophrenia and bipolar disorder [71]. Similarly, the analysis of data from two independent cohorts (the B-SNIP and the New Mexico cohorts) demonstrated that symptom-based approaches account for a larger percentage of variance of real-world functioning compared to DSM categorizations in patients with psychosis [72]. Other analyses of data from the B-SNIP study also clearly show the existence of psychosis biotypes that cross categorical diagnostic constructs (for a review see [73]).

## Utility of additional (bio)markers

It should be noted that a high reliability of schizophrenia diagnosis does not correspond with its validity, which remains low. In this regard, ICD and DSM systems have largely improved diagnostic process and communication between mental health professionals. However, the assessment of specific biomarkers is still recommended to exclude organic causes of psychopathology. Moreover, low validity questions the usefulness of current diagnostic operationalization and signals the necessity of further improvements.

Biomarkers play a significant role in understanding the pathophysiology of both schizophrenia and MS. However, they are still the gold standard only for MS diagnosis [19]. Nevertheless, there is an intensive research activity that aims to improve the diagnostic process of schizophrenia by including specific (bio)markers.

### Polygenic risk score and single nucleotide polymorphism (SNP) heritability

The polygenic risk score (PRS) is calculated by multiplying the number of independent risk alleles (usually restricted to common variants in GWAS) an individual carries by the effect size of each variant, then summing these products across variants. PRS can be used as an early screening tool, that is, as genetic biomarkers, to identify at-risk populations [74].

PRS [75] has been assessed in schizophrenia, and various SNPs have accounted for around 7.5% (median value across cohorts) of the diagnostic variance in the liability scale [76, 77]. Computing SNP heritability has confirmed that part of the risk of developing schizophrenia is captured by common genetic variation, with an estimated SNP heritability of 24% in the largest GWAS to date [78].

For MS, the proportion of phenotypic variation attributable to additive effects of all typed/imputed SNPs across the genome, SNP heritability, was estimated at 19.2% in the most recent GWAS [79]. SNP heritability of schizophrenia and MS are therefore close, but they could also be partly shared. Genes encoding antigen-presenting molecules within the human major histocompatibility complex (MHC), for example, account for the highest component of genetic risk for many diseases, including both MS and schizophrenia [80]. Interestingly, it has been shown that MS shares the involvement of the same human leukocyte antigen (HLA) alleles with schizophrenia, but not with bipolar disorder [14]. However, the authors of this study have found that directionality of the effect might be opposite in case of schizophrenia and MS. Specifically, the DRB1*03:01 and DQB1*02:01 alleles that appeared to increase the risk of MS were found to decrease the risk of schizophrenia. Moreover, the study demonstrated the presence of 20 non-MHC loci that are associated with the risk of both schizophrenia and MS. Another study has further identified 36 loci associated with both disorders and implicated in immune response and B-cell signalling pathways [38].

### CSF and peripheral markers

Some studies intensively explored genetic, proteomic and immune markers to improve diagnostic process in schizophrenia. However, it is likely that most of them can only identify a subset of patients with schizophrenia. Indeed, monogenic causes and polygenic risk scores for schizophrenia explain less than 7% of cases [81]. Moreover, genetic testing in schizophrenia is still not common and usually limited to rare familial cases or genetic syndromes associated with psychosis. However, there is a growing interest in translating epigenetic marks to a diagnostic process of schizophrenia. It has been found that the expression of several miRNA types might reach up to 0.9 value for area under receiver operating characteristic curve (AUC) by plotting sensitivity and specificity [82].

The potential use of proteomic markers is also limited. For instance, a recent meta-analysis of spectrometry-based proteomic studies has demonstrated the upregulation of ficolin-3 together with the downregulation of apolipoproteins (APO1, APOA2, APOC1 and APOC3) in patients with schizophrenia [83]. However, heterogeneity was significant and, as noted by the authors, likely attributable to diagnostic criteria used, comorbidities, characteristics of pharmacotherapy, sociodemographic variables and substance use. Therefore, observed findings might have insufficient sensitivity and specificity. Also, causal association cannot be established due to a lack of longitudinal studies.

Similar shortcomings might be relevant for immune markers. Moreover, it has been observed that most of them, including cytokines and the kynurenic acid pathway markers, show altered levels not only in patients with schizophrenia but also in those with bipolar disorder and major depression [84, 85]. However, some of them might show concordant patterns in the peripheral blood and CSF [85]. It is also likely that the development of several markers will be required to improve diagnostic process in schizophrenia. Indeed, multimodal tests seem to have greater potential; e.g., the serum test based on 51 immunoassays developed by Schwarz et al. [86] reached sensitivity of 83% and specificity of 83% for schizophrenia. An interesting approach was also adopted by Trossbach et al. [87] who used the “reverse-translational approach” to differentiate a subset of schizophrenia patients in whom the blood test reached high sensitivity. Such a method was based on the rat model with overexpressed disrupted-in-schizophrenia 1 (DISC1) protein. The interconnected set of dysregulated immune genes revealed in rats was then tested in patients with schizophrenia and healthy controls. The top two marker genes were able to biologically classify a subset of 27% of schizophrenia patients with a sensitivity of 97% [87].

Conversely, in MS, CSF serves for the exclusion of other diseases, and specifically, CNS-restricted production of immunoglobulins (e.g. oligoclonal bands) aids diagnosis. The oligoclonal bands are considered in a diagnosis of clinically isolated syndromes and when clinical lesions in MRI are disseminated neither in time nor in space [88]. However, oligoclonal bands are not specific due to their occurrence in other inflammatory and autoimmune diseases of the brain. On the other hand, their absence does not exclude disease [89]. Despite the fact that CSF analysis is rarely used in differential diagnosis of schizophrenia, there are cases of changing the initial diagnosis of first-episode psychosis into organic psychosis in the course of MS [90].

### Neuroimaging

The ENIGMA (Enhancing NeuroImaging Genetics through Meta Analysis) consortium has prepared a large-scale analysis of brain morphometry in schizophrenia (more than 5,000 patients), which showed that patients with schizophrenia have smaller hippocampus, amygdala, nucleus accumbens and total cranial volume. Moreover, they had larger pallidum and lateral ventricle volumes. Additionally, their overall cortical surface was smaller and thicker, especially in frontal and temporal regions [91]. Partially, these observations are related to the clinical features of schizophrenia. For instance, a severity of negative symptoms was negatively associated with the volume of prefrontal region, especially the left medial orbitofrontal cortex [92]. Another study has indicated that reduced cortical volume is positively associated with clinical severity [93]. Moreover, spectroscopic studies have revealed higher density of D2/D3 receptors and abnormalities in glutamate levels in several brain regions of patients with schizophrenia [94].

Despite high reproducibility of above-mentioned findings, they are neither sufficiently specific nor sensitive to be used in diagnostic process inter alia due to a genetic overlap of schizophrenia and bipolar disorder, which has been revealed in relation to neural correlates [95, 96]. However, these data are being examined to extract biotypes among patients with schizophrenia spectrum disorders [73]. Currently, the deep learning and machine learning models are extensively used for diagnostic purposes [97, 98]. One of such attempts is the use of fMRI with machine learning to identify functional striatal abnormalities showing high sensitivity (79.0%) and specificity (81.5%) in differentiating patients with schizophrenia and healthy controls [99]. Furthermore, the combination of neuroimaging modalities may achieve a sensitivity of 85.98% and specificity of 87.34% [100], which are slightly higher than in single modalities according to a previous meta-analysis [101].

Also, the perception of schizophrenia as a disorder of dysfunctional connectivity provides important insights into potential underlying mechanisms. Indeed, it is now being increasingly apparent that models considering direct interactions between lesions located in specific brain regions and cognitive or sensorimotor deficits as well as various psychopathological symptoms are not useful in case of schizophrenia [102]. Although region-specific abnormalities can be observed in schizophrenia, they represent the consequence of more global alterations [103]. A recent meta-analysis has revealed significant hypoconnectivity between the seed regions and the auditory network (left insula), the core network (right superior temporal cortex), the default mode network (right medial prefrontal cortex, left precuneus and anterior cingulate cortices), the self-referential network (right superior temporal cortex) and the somatomotor network (right precentral gyrus) in subjects with schizophrenia [104]. However, the authors have found no evidence of significant hyperconnectivity in this population. Importantly, the application of multivariate deep learning techniques to analyse fMRI data has been found to largely improve the prediction of psychiatric diagnosis. For instance, the application of these approaches can increase the accuracy rates of differentiating individuals with schizophrenia and healthy controls up to 92% [105].

On the contrary, neuroimaging is crucial in the diagnosis and future monitoring of MS. Nevertheless, per se, also MRI is not specific as similarly appearing lesions occur in various diseases [106].

### Electrophysiology

Ample evidence shows that abnormalities in the connectivity of brain macro- and microcircuitry are associated with the development of psychosis [107]. In cortical EEG research, signal-to-noise ratios are low, particularly in patients with cognitive decline, due to elevated background noise and diminished signals related to information processing [73]. In parallel, the analysis of microstates in resting-state EEG has been performed with similar accuracy. The EEG indices might also be disturbed in people at a high risk of psychosis and those in early stages of schizophrenia [108]. Partially, they are related to the transition to psychosis in high-risk individuals and poor functional outcome. Large prospective studies are required to verify those findings due to the heterogeneity of previous studies [109]. Similar to other (bio)markers, advanced data science methods on electrophysiology are not sufficient enough to replace a standard diagnosis of schizophrenia due to a lack of appropriate specificity and sensitivity at the moment [110].

In turn, impairment of the function of demyelinated neurons in MS results in abnormal signal conduction, which can be visualized in EEG. Despite a marginal role in diagnostic guidelines, evoked potentials, chiefly visual ones, are instrumental in clinical practice in case of diagnostic uncertainty and monitoring of disease progression [111, 112].

## Improvement of clinical diagnosis in psychiatry and neurology (ICD-11 and DSM-5)

The development of the ICD-11 chapter on mental, behavioural or neurodevelopmental disorders (MBND) and the underlying statistical classification represent the first major revision of the world’s foremost classification of mental disorders in nearly 30 years. Substantial changes have been made to ensure global applicability, clinical utility, scientific reliability and validity in the light of current evidence (World Health Organization, International Classification of Diseases, 11th Revision, 2021).

The chapter on schizophrenia or other primary psychotic disorders with its categories (schizophrenia, schizoaffective disorder, schizotypal disorder, acute and transient psychotic disorder, delusional disorder, and 6A2Y – other specified primary psychotic disorder) demonstrates notable steps towards dimensionality with the introduction of severity-graded (none, mild, moderate, severe, unspecified) symptom specifiers (positive symptoms, negative symptoms, depressive mood symptoms, manic mood symptoms, psychomotor symptoms, cognitive symptoms) for all primary psychotic disorders, as well as longitudinal (first episode, multiple episodes, continuous) and cross-sectional course specifiers (currently symptomatic, in partial remission, in full remission) for some primary psychotic disorders. In comparison with ICD-10, besides the name change for the whole group (using “primary” to indicate the not-yet-fully-known aetiopathogenesis of the group, to rule out origin due to other mental disorders, and to avoid the former incorrect term “non-organic”), further changes of the chapter of psychotic disorders include deemphasis of first-rank symptoms [113] due to their lack of evidence of specificity for schizophrenia (keeping only experiences of influence, passivity or control among the four core symptoms out of which at least one symptom is needed for diagnosis). Out of the total seven essential features, at least two need to be present most of the time for 1 month or more, being no manifestation of another medical condition including disorders due to substance use or withdrawal. Classical schizophrenia subtypes due to a lack of evidence for prospective value and clinical validity have also been omitted.

Accordingly, compared to ICD-10, the pattern of essential symptoms for diagnosis has clearly changed. Although the inter-rater reliabilities for a clinical diagnosis of schizophrenia according to ICD-10 and ICD-11 either by patient-based clinical or by vignette-based internet field studies have been shown to be high [53, 114, 115], with utility ratings being slightly better for ICD-11, epidemiological questions of prevalence differences, stability of caseness in transition from ICD-10 and treatment outcomes for ICD-11-diagnosed cases still need to be studied.

Chapter 6E61 on secondary psychotic syndrome allows for a classification of psychotic syndromes according to their (causally) underlying diseases of known origin using post-coordination (e.g. from 8A40 MS, 8A40.0 relapsing–remitting MS or 8A40.1 primary progressive MS), which may give hints for research on joint pathomechanisms between the two disorders.

The digital ICD-11 platform (World Health Organization, International Classification of Diseases, 11th Revision) with the MMS (Mortality and Morbidity Statistics), including options for complex coding by means of pre- and post-coordination, together with the coding tool allow for going beyond the static categorical diagnosis. The CDDR (Clinical Description and Diagnostic Requirements) for the chapter on psychotic disorders (to be found in the Foundation of the platform) builds on a “content model” with its diagnostic core of “essential” and “additional clinical features” with further information on boundaries with normality and other disorders and conditions, course features, developmental presentations, culture/sex/gender-related features, allowing for a composite categorical and dimensional diagnostic classification with a longitudinal and cross-sectional type of course staging and symptom profiling. Thereby, due to the much broader spectrum of diagnostic features, clinicians will be enabled to apply more individualized treatment and care [116]. Changes in the diagnostic guidelines from ICD-10 to ICD-11 are reflecting the ongoing developments in nosological science. This will be further supported by the Maintenance and Proposal tools allowing for ongoing modifications of the classification according to empirically based rational and relevant commentaries or proposals for change, hence for ongoing improvement of the diagnostic classification as kind of a “living” diagnostic classification – supervised and guided by respective WHO Family of International Classifications expert committees.

The chapter on psychotic disorders of ICD-11 did not introduce a “paradigm shift” – similar to DSM-5 [117], since a neurobiological concept could not yet be implemented. After more than 110 years, Bleuler’s concept of “the schizophrenias” [118] with the heterogeneity of prognosis and outcome indirectly paved the way for modifications of the concept. All these developments build on aiming for classificatory innovations [119] of either deconstructing psychosis, transdiagnostic hierarchical “reconstruction” explaining comorbid or overlapping disorder constellations - based on psychopathological symptom network theories and dimensional concepts - like the Hierarchical Taxonomy of Psychopathology (HiTOP), or the general psychopathology (p-)factor explaining the co-occurrence of symptoms across various disorders. Another very basic transdiagnostic approach as the US NIMH initiative on Research Domain Criteria (RDoC) is a research framework providing a neuropsychobiological concept for analysing potential building blocks of mental disorders.

Vis-à-vis these developments, the revised schizophrenia construct in ICD-11 is still alive and may even improve contemporary clinical practice globally. It has salvaged the construct for clinical practice and further “living” revisions until new approaches might be demonstrating their superiority in validity (and utility), stimulating the development of superior versions of treatment and care in correspondingly adapted mental healthcare systems.

It would take time to manage transition, implementation and adaptation of a new construct to be evaluated on top of, and in comparison with, the current classification version during which diagnostics, treatment and care according to the then ICD revision in place need to continue [120]. In conclusion, the new ICD-11 classification for schizophrenia or other primary psychotic disorders and their accompanying diagnostic guidelines will represent an important advance for the field.

In neurology, the revision of ICD-10 has brought significant advances in many fields including epilepsy and headache [121]. The most important progress was the classification of cerebrovascular diseases and neurological disorders in ICD-11 [121]. The immediate consequence of these changes is a better estimation of the burden of neurological disorders, which are estimated today to affect at least one out of three in the general population and are today recognized among the three most common causes of disability and mortality [122, 123].

## Discussion and future prospects

There are several similarities of schizophrenia and MS, mainly in terms of environmental risk factors, the involvement of immune-inflammatory mechanisms, genetic backgrounds, age of onset and the course of illness. However, these similarities need to be interpreted with caution, especially with respect to specific environmental risk factors that might also increase the risk of other mental disorders. Also, results of studies investigating specific neurobiological mechanisms in schizophrenia and MS might be biased by medication effects related to the use of antipsychotics or glucocorticoids that might alter immune-inflammatory responses. However, the main difference of importance in clinical practice is related to the diagnostic process. Indeed, the use of specific (bio)markers is a gold standard in MS, but not in case of schizophrenia. Moreover, while the current diagnostic classifications have abandoned the necessity to indicate the subtypes of schizophrenia, subtyping of MS is still recognized as one of important points in the diagnostic process. Moving the field forward will require a better understanding of symptoms defining what we call schizophrenia and their biological underpinnings.

The current diagnostic systems of mental disorders definitely improved reliability of diagnosis, including that of schizophrenia; however, validity still leaves much to be desired [124]. Imagining the future of schizophrenia, its classification and still unknown aetiology and link to immunity, it is not a far-fetched assumption to claim that diagnosing, treating and preventing schizophrenia will undergo a profound change. It is likely that diagnostics in schizophrenia will evolve into subcategories with quantitative trait measures and objective biological markers [65]. This will make it easier for clinicians to prescribe the most effective medication as well as to provide a more accurate prognostic assessment that appears to be valid in several medical specialties, especially oncology [125]. One of the biggest hurdles is to combine the findings from different research areas. There are probably several biological pathways that eventually lead to the current clinical phenotype known as schizophrenia today [126, 127]. Indeed, multiplicity of risk factors (e.g. copy number variations, childhood trauma and substance abuse) and their biological correlates might suggest interindividual variability in underlying aetiologies. However, the heterogeneity of schizophrenia also lies in several clinical aspects, including psychopathological manifestations, course of illness, response to treatment and functional outcomes. This might also explain why specific genes and biomarkers could not be indicated so far, since we might generate too much noise by mixing different etiologic and clinical manifestations. Specifically, unsupervised algorithms could help researchers identify yet unknown biomarker-driven schizophrenia endo-phenotypes. These data-driven approaches can only be successfully applied and deployed if clinical expertise and profound knowledge of the underlying statistical methods are combined.

**Figure 1.**
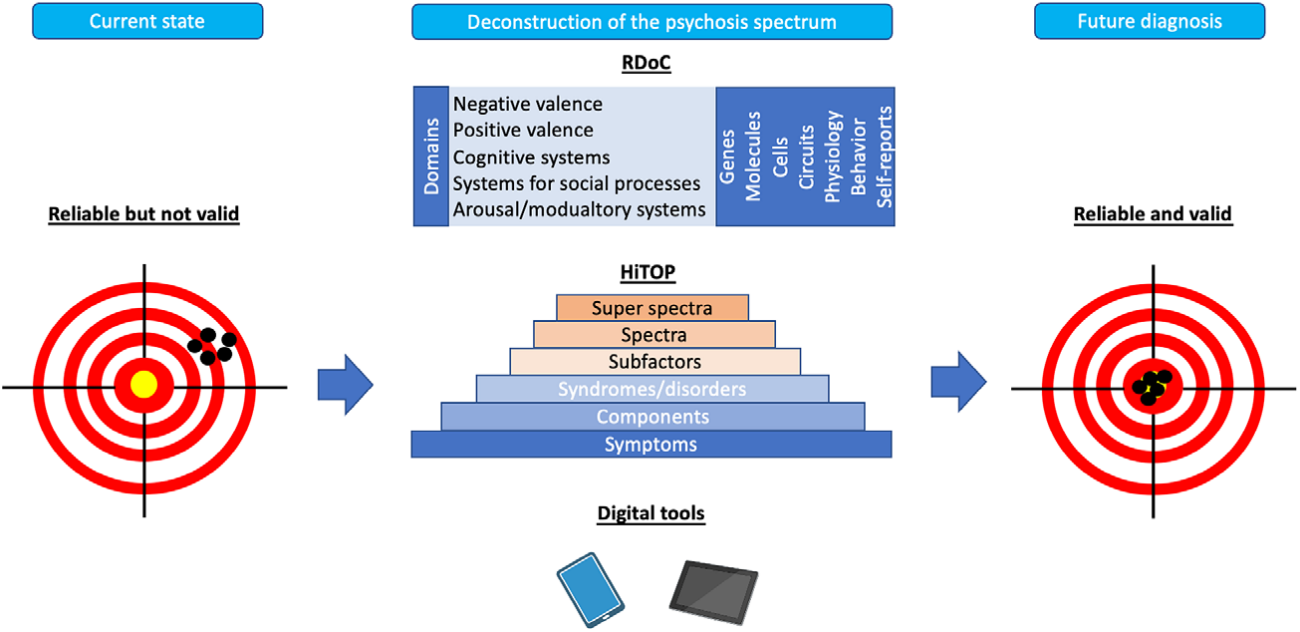
Overview of future directions for the field of clinical diagnosis of schizophrenia. Current diagnostic criteria of schizophrenia are characterized by sufficient reliability but low validity. The figure shows directions for the field that focus on the deconstruction of psychosis spectrum in order to improve validity. This process can be approached through novel interfaces, including the Hierarchical Taxonomy of Psychopathology (HiTOP) and the Research Domain Criteria (RDoC) as well as digital phenotyping.

The deconstruction of the psychosis spectrum through the insight into the dimensions of psychopathology might serve as one of the most promising approaches to increase the validity of schizophrenia diagnosis (Figure 1). For instance, there is evidence that a five-factor structure of psychopathology (positive and negative symptoms, disorganization, manic and depressive symptoms) appears not only in patients with schizophrenia but also in those with affective psychosis [128]. Also, it has been shown that certain cognitive deficits, e.g., working memory impairments, are more closely related to psychosis dimension than the current diagnostic categorizations [129]. Finally, common risk factors of schizophrenia might also contribute to the development of other mental disorders. Targeting the dimensions of psychopathology to improve validity will require deep phenotyping covering known environmental risk factors and biomarkers in large cohorts. This approach can include the multiplicity of known environmental exposures that increase the risk of psychosis within single scores. For instance, it has been shown that the “polyenviromic risk score” – covering cannabis use, urbanicity, season of birth, paternal age, obstetric and perinatal complications and a history of childhood trauma – can predict conversion to overt psychosis in people at a high risk of familial psychosis [130]. The deconstruction of the psychosis spectrum can further operate through interfaces that were developed to improve psychiatric nosology, such as the RDoC and the HiTOP. RDoC aims to refine the diagnostic criteria by investigating biobehavioural dimensions [131]. It has been developed by the National Institute of Mental Health (NIMH) and highlights the existence of specific domains of functioning (negative and positive valence systems, cognitive systems, systems for social processes as well as arousal/modulatory systems) that are known to be impaired in mental disorders. According to the RDoC assumptions, each domain is characterized by the underlying neurobiological substrates that can be studied to provide translational perspectives. These neurobiological substrates can be explored within specific units of analysis (i.e. genes, molecules, cells, circuits, physiology, behaviour and self-reports). In turn, HiTOP addresses the heterogeneity of psychopathology according to their observed covariation and co-occurrence without insights into underlying neurobiology [132]. It is based on the hierarchical structure where symptoms cluster within the higher-order clusters operationalized within components, syndromes, subfactors, spectra and super spectra. The identification of hierarchical structure can be obtained by factor analysis and other related analytical approaches. It has also been proposed that combining both interfaces may further improve psychiatric nosology [133].

Progress in the field of clinical diagnosis of schizophrenia spectrum disorders might also be achieved through the use of novel digital tools. Over the past decades, the use of digital tools in medicine has constantly grown, heralding a new era also in psychiatry. Some new digital tools have become rapidly a common standard (e.g. the use of electronic medical records), others are constantly gaining momentum (e.g. telepsychiatry and digital therapies), while some others are still in their nascent phase (e.g. virtual reality and artificial intelligence) [134]. More in detail, for psychiatric diagnosis, a recent systematic review has reported that, to date, most of the studies adopting digital tools for diagnostic purposes actually employed digitized versions of standard questionnaires, mostly to deal with affective syndromes, with variable accuracy and significant risk of bias [135]. The constant, passive and massive accumulation of data from smartphones, however, provides a unique opportunity to capture longitudinal and multimodal health-related data. These approaches might reveal particularly useful to timely capture the psychopathology of early stages of psychosis, fostering early management and recovery [136]. Moreover, the use of digital phenotyping techniques may greatly help in clinical monitoring over time by recording a variety of integrated moment-by-moment assessments of individual phenotypes using data from mobile and connected technologies [137, 138]. The evolution of this direction holds a significant potential in both diagnostic and therapeutic terms, but it still requires larger samples, better quality of data, the development of standardized analytical procedures and the evaluation of ethical concerns [139]. The added value of digital approaches to the diagnosis and management of schizophrenia will probably further increase when the combination of variables related to psychopathology and behaviour with those that capture neuroimaging findings, peripheral blood markers and genetic background will be achieved [137].

In sum, the ongoing progress in clinical neurosciences has provided new perspectives for a better understanding of processes underlying the pathophysiology of schizophrenia. Combining a variety of already proposed approaches to address clinical and etiological heterogeneity across the psychosis spectrum might help to dissect valid diagnostic constructs. Translation of approaches from other disciplines and medical specialties, including neurology, might further improve the diagnostic process in psychiatry. For clinical practice, the intersection between psychiatry and neurology indicates the necessity to follow routine collaboration of both disciplines in several clinical situations. This might be of particular importance in case of diagnosing psychotic disorders when organic causes need to be ruled out or in case of emergent psychotic symptoms in the course of diagnosed neurological disorders, including MS. Finally, it is necessary to evoke the social context and indicate the importance of listening to patients and their relatives in shaping the ease and use of future diagnoses.
